# Supplementary material for: Antimicrobial Properties of Different Hop (Humulus lupulus) Genotypes
Source: Plants (Basel). 2022 Dec 26;12(1):120. doi: 10.3390/plants12010120 (PMC9824274; doi:10.3390/plants12010120)

Plants

Supplementary Material

Antimicrobial Properties of Different Hop (*Humulus lupulus*) Genotypes

Zala Kolenc[1], *Tomaž Langerholc*[2], *Gregor Hostnik*[1], *Miha Ocvirk*[3], *Sara Štumpf*[1], *Maša Pintarič*[2], *Iztok Jože Košir*[3], *Andreja Čerenak*[3], *Alenka Garmut*[2] *and Urban Bren*[1,4,5]*

[1] Laboratory of Physical Chemistry and Chemical Thermodynamics, Faculty of Chemistry and Chemical Technology, University of Maribor, Smetanova ulica 17, SI-2000 Maribor
[2] Department of Microbiology, Biochemistry, Molecular Biology and Biotechnology, Faculty of Agriculture and Life Sciences, University of Maribor, Pivola 10, 2311 Hoče, Slovenia
[3] Department for Agrochemistry and Brewing; Department for Plants, Soil and the Environment, Slovenian Institute of Hop Research and Brewing, Cesta Žalskega tabora 2, 3310 Žalec, Slovenia
[4] Department of Applied Natural Sciences, Faculty of Mathematics, Natural Sciences and Information Technologies, University of Primorska, Glagoljaška ulica 8, SI-6000 Koper, Slovenia
[5] Institute of Environmental Protection and Sensors, Beloruska ulica 7, SI-2000 Maribor, Slovenia

* Correspondening Author
E-mail addres: urban.bren@um.si
tel.: +386-2-229-4421.

Additional xanthohumol identity confirmation of compound was obtained using 1H NMR and LC/MS spectroscopy. 1H spectrum (300 MHz, $(CD_3)_2SO$, d/ppm, J/Hz) δ = 14.60 (1H, s, 2'-OH), 10.55 (1H, s, 4-OH), 10.02 (1H, s, 4'-OH), 7.74 (1H, d, J =15.5 Hz, Hα C=O), 7.64 (1H, d, J = 15.6 Hz, Hβ C=O), 7.55 (2H, d, J = 8.6 Hz, H-2, H-6), 6.82 (2H, d, J = 8.6 Hz, H-3, H-5), 6.06 (1H, s, H-5'), 5.11 (1H, t, J = 7.1 Hz, H-2''), 3.85 (3H, s, O-Me), 3.12 (2H, d, J = 7.0 Hz, H-1''), 1.68 (3H, s, H-4''), 1.59 (3H, s, H-5'').

Figure S1: Comparison of the experimental data and the model curve of $\ln(OD_{595}/OD_{595,0})$ of *Staphylococcus aureus* inoculated in **100 % MHB** medium for the **HAE Styrian Eagle** extract. In figure the growth curve for A) control sample, B) sample concentration of 3.69 µg/mL, C) sample concentration of 7.80 µg/mL, D) sample concentration of 15.60 µg/mL are presented.

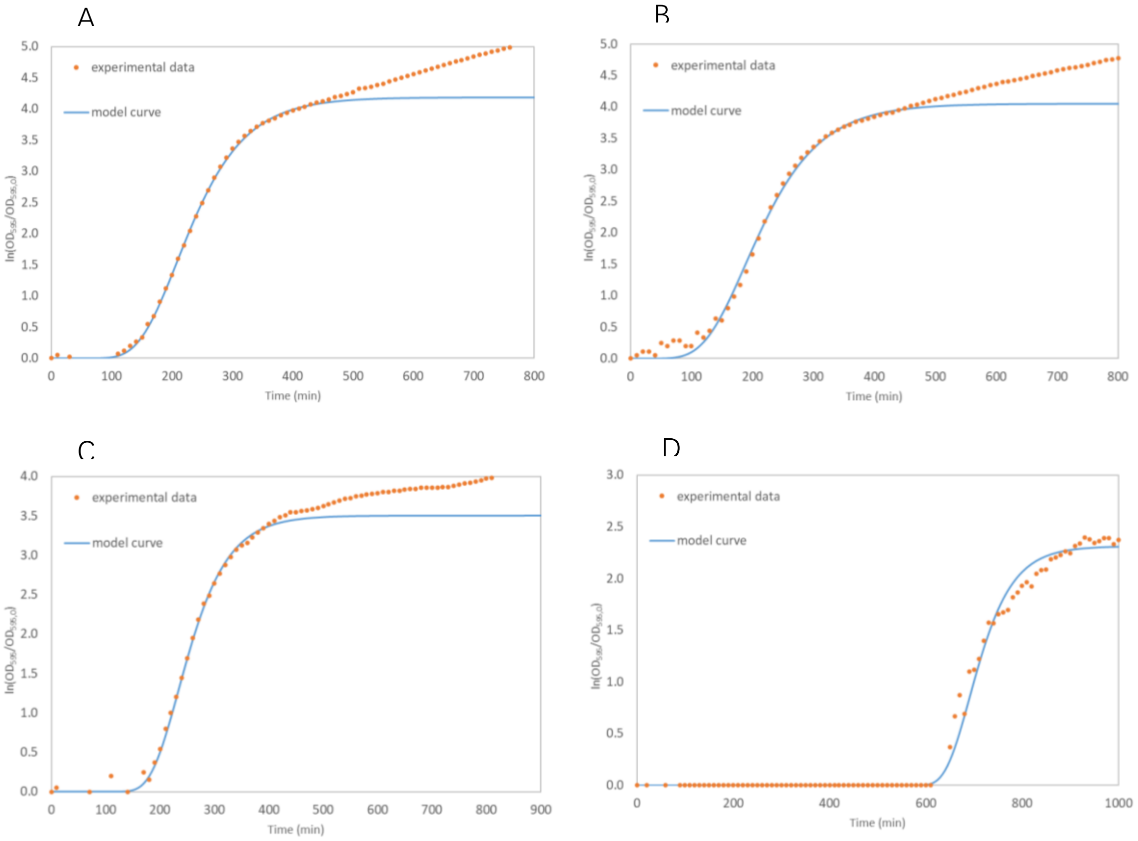

Figure S2: Comparison of the experimental data and the model curve of ln(OD_{595}/$OD_{595,0}$) of *Staphylococcus aureus* inoculated in **100 % MHB medium for the αβ – AF** extract. In figure the growth curve for A) control sample, B) sample concentration of 4.40 µg/mL, C) sample concentration of 6.58 µg/mL, D) sample concentration of 9.88 µg/mL, E) sample concentration of 14.80 µg/mL ,F) sample concentration of 22.20 µg/mL are presented.

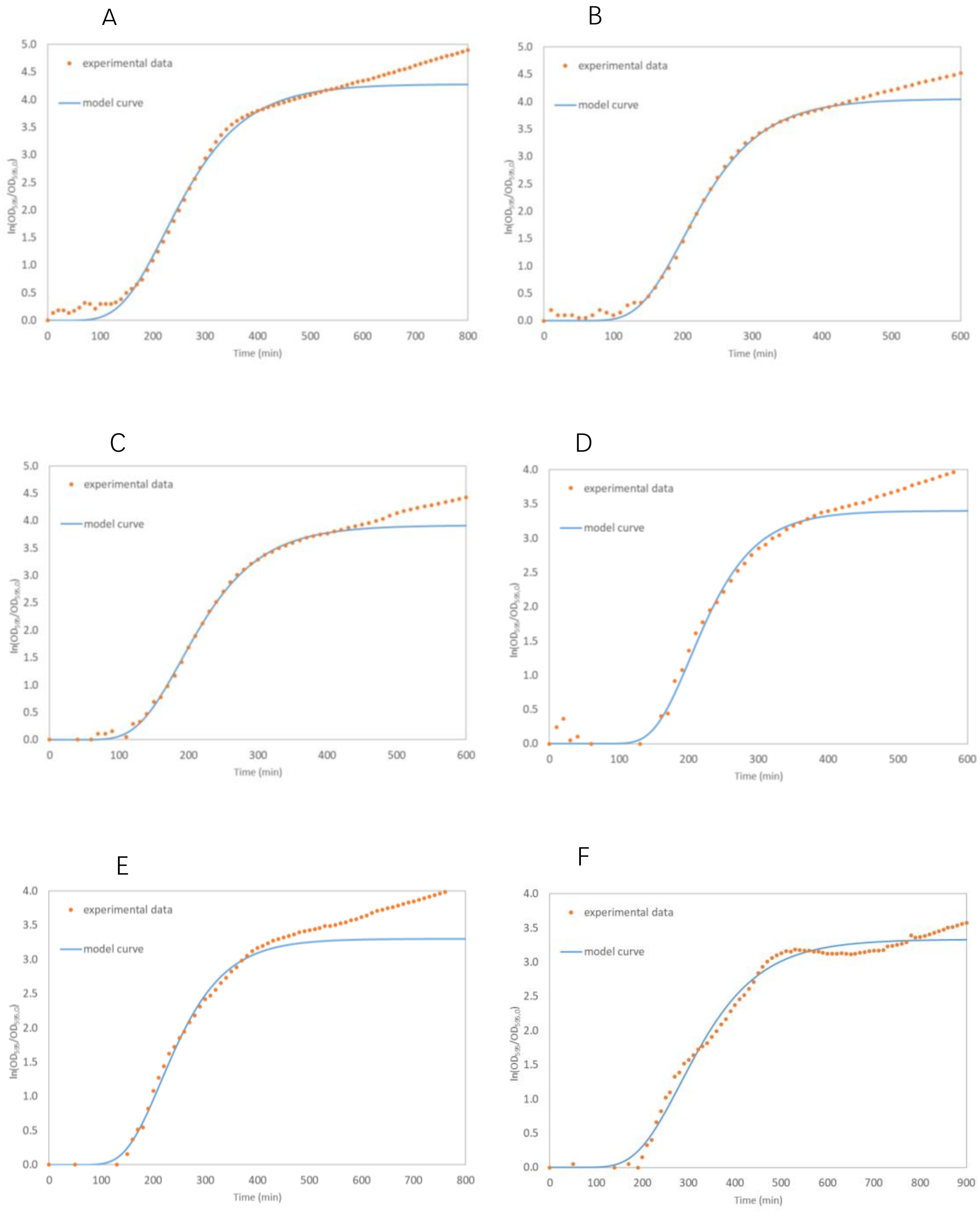

Figure S3: Comparison of the experimental data and the model curve of ln($OD_{595}/OD_{595,0}$) of *Staphylococcus aureus* inoculated in **100 % MHB medium for the β – AF** extract. In figure the growth curve for A) control sample, B) sample concentration of 1.76 µg/mL, C) sample concentration of 2.63 µg/mL, D) sample concentration of 3.95 µg/mL, E) sample concentration of 5.93 µg/mL are presented.

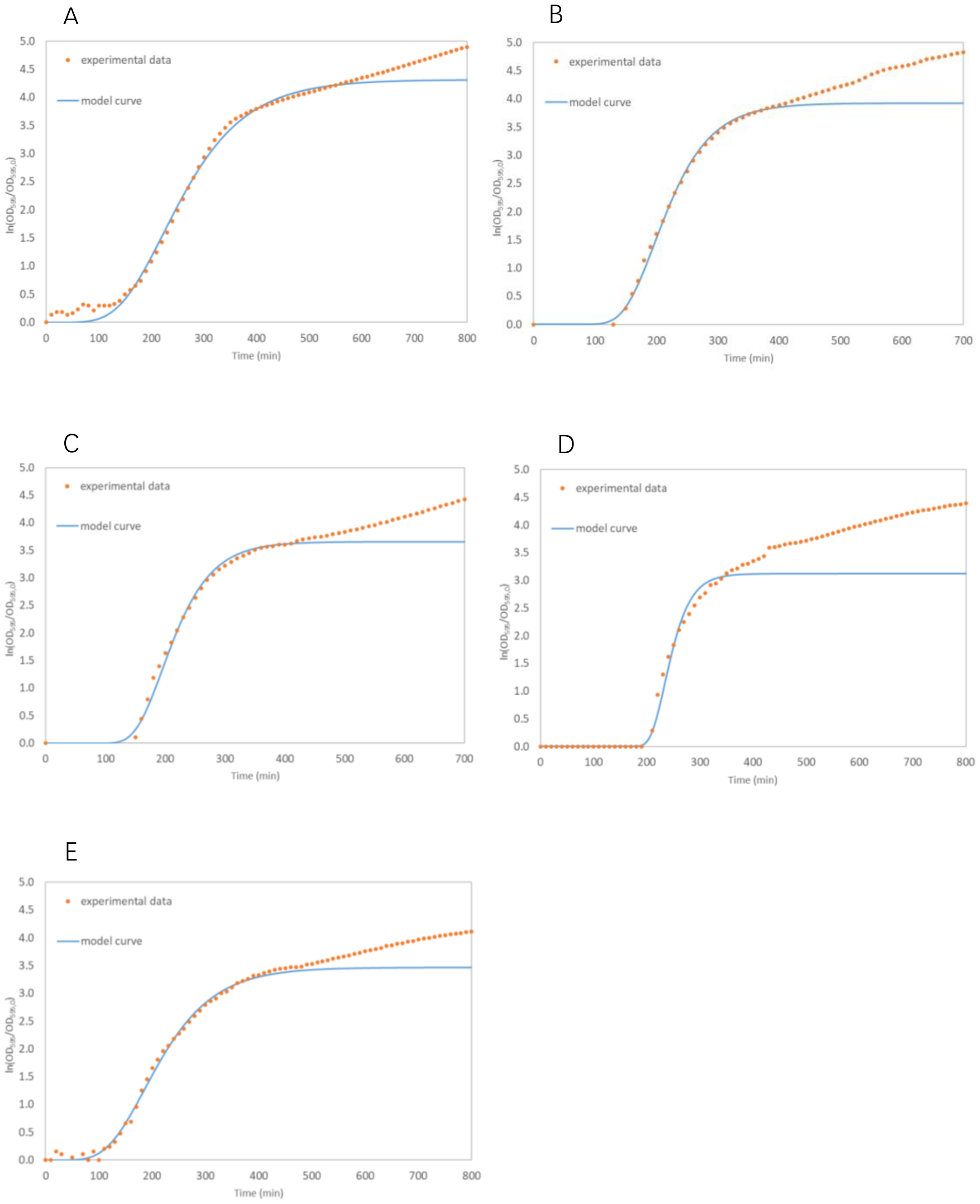

Supplement: Supplementary file 1 [file plants-12-00120-s001.zip › plants-2087727-supplementary.pdf]
